# Cerebral Changes Following Carpal Tunnel Syndrome Treated with Guided Plasticity: A Prospective, Randomized, Placebo-Controlled Study

**DOI:** 10.1055/s-0044-1792169

**Published:** 2024-11-14

**Authors:** Magnus Flondell, Peter Mannfolk, Birgitta Rosén, Isabella M. Björkman-Burtscher, Anders Björkman

**Affiliations:** 1 Department of Hand Surgery, Skåne University Hospital, Malmö, Sweden; 2 Department of Hand Surgery, Translational Medicine, Lund University, Malmö, Sweden; 3 Department of Medical Imaging and Physiology, Skåne University Hospital, Lund, Sweden; 4 Department of Radiology, Institute of Clinical Sciences, Sahlgrenska Academy, University of Gothenburg, Gothenburg, Sweden; 5 Department of Radiology, Sahlgrenska University Hospital, Region Västra Götaland, Gothenburg, Sweden; 6 Department of Hand Surgery, Sahlgrenska University Hospital, Gothenburg, Sweden; 7 Department of Hand Surgery, Institute of Clinical Sciences, Sahlgrenska Academy, University of Gothenburg, Gothenburg, Sweden

**Keywords:** carpal tunnel syndrome _1_, magnetic resonance imaging, somatosensory cortex, anesthesia, hand

## Abstract

**Background** Compression neuropathy, such as carpal tunnel syndrome (CTS), results in changed afferent nerve signaling, which may result in changes in somatosensory brain areas. The purpose of this study was to assess cerebral changes following unilateral CTS and to assess short-term and long-term cerebral effects of guided plasticity treatment using ipsilateral cutaneous forearm deafferentation.

**Methods** Twenty-four patients with mild-to-moderate unilateral CTS were randomized to treatment with anesthetic cream (EMLA) or placebo. Patient-rated outcomes were assessed using Boston CTS questionnaire and disability of arm, shoulder, and hand questionnaire (QuickDASH). Patients were assessed for tactile discrimination and dexterity. Cortical activation during sensory stimulation was evaluated with functional magnetic resonance imaging at 3T. Assessments were performed at baseline, 90 minutes, and 8 weeks after treatment.

**Results** Functional magnetic resonance imaging showed that sensory stimulation of the hand with CTS resulted in significantly less cortical activation in the primary somatosensory cortex (S1) than stimulation of the healthy hand. Treatment with cutaneous forearm deafferentation on the side with CTS resulted in increased cortical activation in S1 both after the initial treatment and following 8 weeks of treatment. In addition, QuickDASH and tactile discrimination showed improvement in the EMLA group over time.

**Conclusions** Stimulation of median nerve-innervated fingers in patients with unilateral CTS results in smaller-than-normal activation in the contralateral S1. Cutaneous forearm anesthesia on the side with CTS results in larger activation in S1, suggesting recruitment of more neurons, and a slight improvement in sensory function.

## Introduction

Carpal tunnel syndrome (CTS), where the median nerve is compressed at the wrist, is the most common nerve entrapment, with a prevalence of 4 to 8% in the general population. 1 In patients with persisting symptoms, the treatment of choice is surgical decompression of the median nerve, carpal tunnel release (CTR). In patients with mild symptoms, a short duration night splint and activity modification may be sufficient. It has been estimated that 2 to 4% of the general population undergo CTR during their lifetime. 2 3 4 5 CTR is generally believed to relieve symptoms, however, unsatisfactory symptom relief is not uncommon. 6 7 8 9 Reasons for a suboptimal outcome of CTR are: incomplete decompression of the nerve, concomitant diseases (diabetes, hypothyroidism), concomitant vibration-induced neuropathy, or wrong diagnosis. 6 7 8 10 11

It is well known that a median nerve injury and repair results in changed afferent nerve signaling, and secondary to this, structural and functional changes in the central nervous system (CNS). 12 13 Similar changes have been suggested in patients with CTS and the functional deficits seen in patients with CTS have been suggested to reflect reorganization in somatosensory areas in the brain. 14 15 16 17

Guided plasticity is a concept where the dynamic capacity of the brain is used, for therapeutical purposes, to replace or improve damaged functions. 18 19 To our knowledge, there is no prior study that has used guided plasticity to treat patients with CTS and to demonstrate potential cerebral changes using functional magnetic resonance imaging (fMRI). However, one prior study has used acupuncture on patients with CTS. 20 This placebo-controlled study on patients with bilateral CTS showed similar symptom relief in both groups, whereas only the group treated with acupuncture improved in neurophysiological outcomes. 20 An example of guided plasticity is cutaneous forearm deafferentation where the skin of the volar forearm is anesthetized with an anesthetic cream. This results in rapidly improved sensibility in the fingers in healthy volunteers 21 as well as in patients with impaired sensation due to nerve injury or vibration-induced neuropathy. 22 23 24 25 The mechanism behind this improved sensibility is thought to be based on recruitment of more neurons in the primary somatosensory cortex. 26 27

## Methods

The aim of this study was to assess cerebral changes in patients with mild to moderate unilateral CTS and to assess cerebral short- and long-term effects of treatment using ipsilateral cutaneous forearm deafferentation in patients with CTS.

Patients who had been referred to the Department of Hand Surgery, Skåne University Hospital, Malmö, Sweden due to suspected CTS were screened for participation. Inclusion criteria were: unilateral subjective symptoms of CTS for more than 3 months, classic or probable CTS according to Katz hand diagram, 2 28 age between 18 and 70 years, and a nerve conduction study (NCS) with a fractionated sensory nerve conduction velocity for the median nerve across the wrist of 40 m/s or less on the affected side and of more than 43 m/s on the contralateral side, as well as no contraindications for MR examinations. Exclusion criteria were bilateral symptoms, having been operated for CTS previously, prior wrist or carpal fracture, diabetes, thyroid disease, rheumatoid arthritis, neurological disease, drug abuse, complete conduction block on electroneurography (ENG), or prior regular exposure to hand-held vibrating tools. Participants should be able to read and understand Swedish and to be able to fill out the patient-rated outcome measures and the informed consent in a proper way. The participants comprise all eligible patients with unilateral clinically and neurographically confirmed CTS referred for 4 years. 27 Clinical assessment and study inclusion were performed by a senior consultant in hand surgery. Clinical testing of outcome parameters, training instructions, and treatment instructions were given by a senior consultant in hand surgery and/or an experienced occupational therapist. A technician from the Department of Clinical Neurophysiology performed the NCS examinations, which were interpreted by a senior consultant in neurophysiology. All were blinded to treatment randomization.

Participants were randomized to treatment with either 15 g of a local anesthetic cream containing 2.5% lidocaine and 2.5% prilocaine (EMLA; AstraZeneca AB, Södertälje, Sweden) or with a placebo cream. The placebo cream was visually and cosmetically identical to EMLA and did not include any anesthetic drugs. EMLA or placebo was applied to the volar aspect of the forearm, in an area from the wrist and 15 cm proximal on the same side as the CTS for 90 minutes. The initial treatment was done at the hospital, after that the participants followed a treatment protocol shown beneficial in patients operated with median nerve repair. 24 In this protocol, participants administered 15 treatments themselves at gradually increasing intervals for 8 weeks. In addition, both groups were instructed to perform a standard sensibility training program on a daily basis.

### Clinical Assessment

CTS was rated according to Padua 29 (grade 1 = extreme; grade 2 = severe; grade 3 = moderate; grade 4 = mild; grade 5 = minimal; grade 6 = negative).

Subjective symptoms and activity limitations were assessed at baseline and after 8 weeks of treatment using the symptom severity scale (SSS) from the Boston carpal tunnel syndrome questionnaire (BCTQ) 4 28 30 and the short version of the “disability of arm, shoulder, and hand questionnaire” (QuickDASH). 31

Direct assessments focusing mainly on sensory functions were performed in both hands at baseline, directly after the first treatment, and at the 8-week follow-up. During sensory testing, vision was occluded with a screen and the hand being tested was resting comfortably in a supine position.

Clinical testing of the sensory hand function at all three time points (clinical evaluation [CE] 1, 2, and 3) included: two-point discrimination (2PD) according to the “Moberg method” 32 at fingertip level of digits II and V for assessment of tactile discrimination (tactile gnosis) using a single blunted stainless steel pin, and pairs of them, with a diameter of 300 µm, mounted on two separate discs to allow easy switching between the pins' distances (0.7, 1.0, 1.3, 1.6, 1.9, 2.2, 2.5, 2.8, 3.1, 3.4, 4.0, 4.3, 4.6, and 5.0 mm) 21 ; and the Purdue pegboard test for finger dexterity and speed 33 using the right hand or left hand subtest and calculating the mean score of three consecutive trials.

**Fig. 1:**
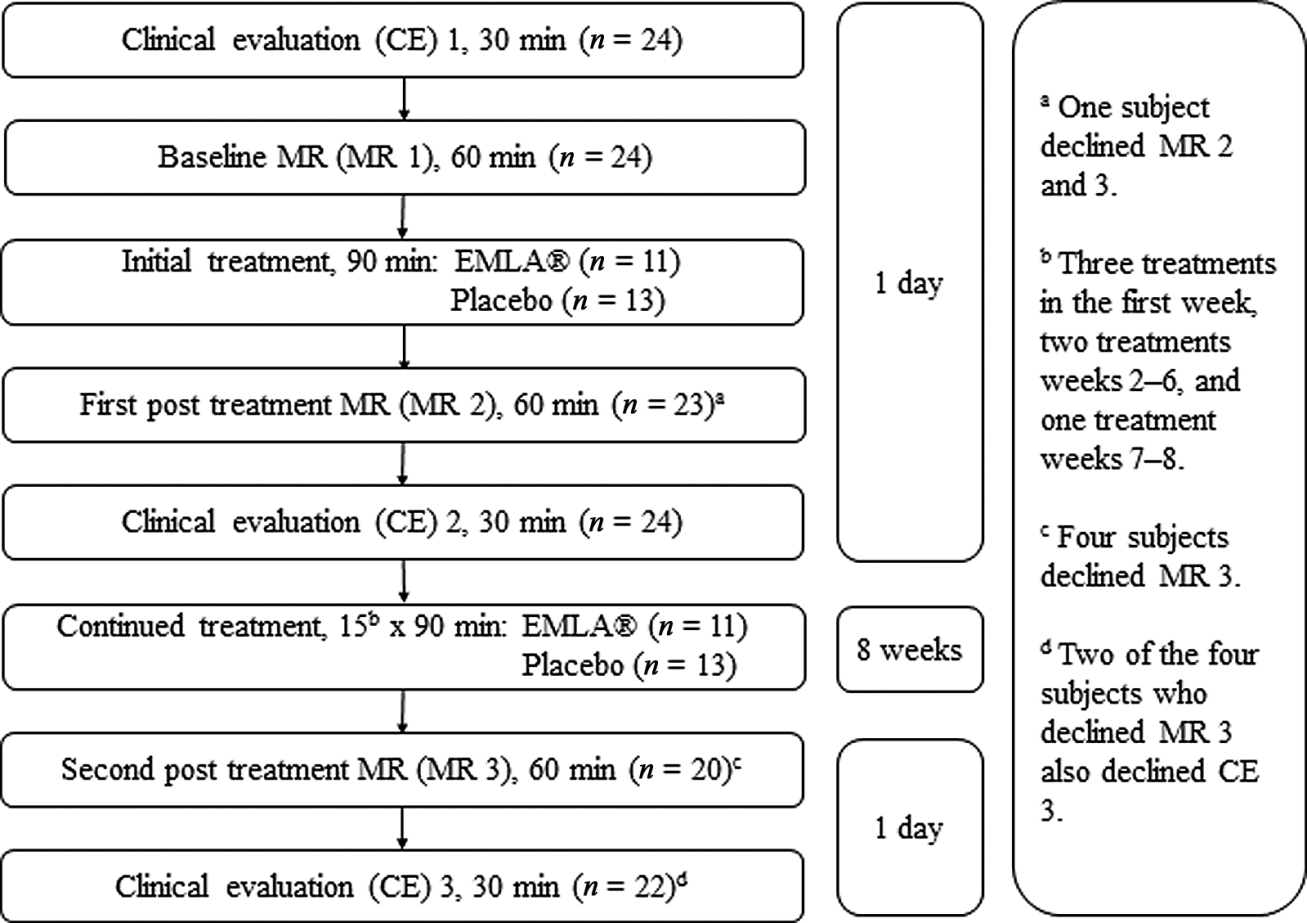
Study setup and time points for clinical evaluation (CE), treatment intervention, and functional magnetic resonance (fMRI) examinations (MR); *n* = number of study participants. EMLA and placebo treatment during the 8-week period was supplemented by patient-administered sensory training sessions in both groups.

Fig. 1 illustrates the timeline of CEs, treatment interventions, and fMRI assessments (MR) in a flow chart.

### Functional Magnetic Resonance Imaging

#### Data Acquisition

fMRI was performed at baseline (MR 1), after the first treatment (MR 2), and after 8 weeks (MR 3) to investigate cortical activation during tactile stimulation of the fingers using a whole-body 3T scanner (MAGNETOM Skyra, Siemens Healthineers, Erlangen, Germany) equipped with a 20-channel head coil.

#### Task Specification

Tactile stimulation was administered using a pneumatically driven and electronically controlled system to ensure precise and reproducible finger stimuli. 34 The device had six individually controlled channels, each consisting of a pneumatic valve (Festo, Germany) connected by a plastic tube (0.8 cm ^2^ ) to a chamber with a membrane (4-D Neuroimaging, San Diego, California, United States). The chambers were applied to the finger pulps of the thumb (digit I), index finger (digit II), and little finger (digit V) of both hands. Stimulation was intended to resemble touch but well below forced touch or pain. The stimulation system was tested in dry runs before each experiment.

#### Design Specification

Tactile stimulation was administered in a randomized order, and randomization order was documented. Tactile stimulation was administered to both median nerve-innervated skin areas (digit I and digit II) and ulnar nerve-innervated skin areas (digit V). Patients were told to rest both arms comfortably on cushions to prevent errors caused by motion. Tactile stimulation of the fingers was performed in a block design, alternating between stimulation and resting condition (100 milliseconds pulse, 1 Hz pulse frequency, 2.5 bars pressure), resulting in four blocks of stimulation for each finger in a classic boxcar function. The duration of each activation/rest block was 17.5 seconds. A gradient echo echo-planar imaging pulse sequence was used for functional imaging. Pulse sequence parameters were TR = 2,500, TE = 30 milliseconds, flip angle = 90°, voxel size = 2 × 2 × 2 mm ^3^ , 33 slices, and 336 dynamic scans.

#### Details on Subject Samples

To facilitate statistical group analysis, functional data from subjects with CTS on the left side ( *n* = 3) were flipped left–right prior to preprocessing and analysis, a strategy that has been used before. 35

Data representing the hand affected by CTS are referred to as affected side/hand or ipsilateral to CTS and data representing the hand not affected by CTS are referred to as healthy side/hand or contralateral to CTS.

#### Data Preprocessing

Preprocessing and analysis of brain imaging data sequences were made using the SPM software package (FIL Methods Group, UCL 12 Queen Square, London, United Kingdom) for MATLAB (Mathworks, Natick, Massachusetts, United States). Evaluation of fMRI data was performed with the SPM12 toolbox for MATLAB. The fMRI data were motion-corrected to the first volume; slice timing was corrected to slice number one.

#### Smoothing and Registration

The fMRI data were subsequently spatially smoothed with a 4-mm smoothing kernel and subsequently normalized to standard space using 2 × 2 × 2 MNI template (Montreal Neurological Institute) from SPM12. 36

#### Statistical Modeling and Statistical Interference

Contrasts used were interaction contrasts of the stimulated fingers. Analyses represent a combination of median nerve-innervated digits (digits I and II) and ulnar nerve-innervated digit V. Activation maps were created with the general linear model, using the SPM12 canonical hemodynamic response function and corrected for serial correlations. 37 The resulting activation maps were visually inspected and evaluated at a statistical threshold of *p* < 0.01, uncorrected for multiple comparisons, to avoid cluster size bias. 38

### Electroneurography

Sensory electroneurography was performed bilaterally at baseline and after 8 weeks, using a Nicolet Viking Select equipment (Nicolet Biomedical Inc., Madison, Wisconsin, United States). The patient's skin temperature was kept above 30°C during the examination. Sensory fibers were stimulated in the thumb, the index finger, and the long finger for the median nerve. Ring electrodes were placed at the proximal interphalangeal and distal interphalangeal joints for the index and long fingers, and for the thumb, just proximal and distal to the interphalangeal joint. Recording electrodes were placed over the median nerve at the proximal wrist crease. In addition, measurements were done on the ulnar nerve. Distal motor latency (ms) and sensory conduction velocity (m/s) were measured according to guidelines. 39

### Statistics

Analyses of changes regarding hand function within and between the two groups at baseline and of longitudinal changes 90 minutes after the first treatment and after 8 weeks of treatment were performed for fMRI data and clinical data. Primary clinical outcome was longitudinal changes in tactile discrimination (2PD) within the two groups between baseline, 90 minutes after first treatment, and results after 8 weeks of treatment. Secondary outcomes are dexterity, SSS, and QuickDASH. Differences between EMLA and placebo group at different time points (CE 1, 2, and 3) were calculated with the Mann–Whitney U-test. Longitudinal analyses in the EMLA group and the placebo group, respectively, were performed using the Wilcoxon signed-rank test. Differences between the EMLA group and the placebo group, when comparing baseline and results after the first treatment and when comparing baseline and results after 8 weeks, were calculated with the Mann–Whitney U-test. *p* -Values ≤0.05 were considered significant.

### Trial Registration

The study was approved by the Swedish Ethical Review Authority (DNr 269–2008, amendment 23–2011). The study is registered in Clinicaltrials.org ID NCT06016049.

### Randomization

The patients were randomized by the investigator who did not perform the clinical follow-up examinations. It was done by assigning patients to one out of two study groups, by retrieving a study number corresponding to a treatment from a computer-generated randomization list.

## Results

Subject demographics are given in Table 1 . Due to a randomization error, one patient received placebo instead of EMLA, thus 13 subjects were included in the placebo group and 11 in the EMLA group. Except that women were overrepresented in both groups, demographics did not differ significantly between groups.

**Table 1:** Cohort demographics

Demographic parameter	Cohort		
	All	EMLA	Placebo
Number of subjects, *n*	24	11	13
Gender, *n* subjects, male/female	4/20	2/9	2/11
Hand affected, *n* subjects, right/left	21/3	10/1	11/2
Age, median (range), years	48 (33–68)	55 (37–66)	43 (33–68)
CTS classification according to Padua a			
Grade 3 (moderate), *n* subjects	17	10	10
Grade 4 (mild), *n* subjects	7	1	3

Abbreviation: CTS, carpal tunnel syndrome.

a Padua classification. 29

### Clinical Assessment

**Fig. 2:**
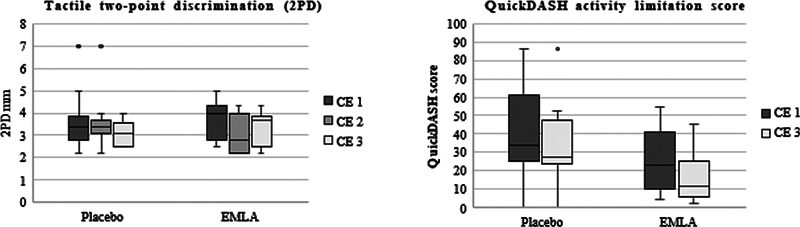
Visualization of EMLA and placebo cohort data for 2PD and QuickDASH at clinical evaluation (CE) time points 1 to 3.

Median data for patient-reported outcome measures, BCTQ and QuickDASH, tactile discrimination measurements with 2PD, and dexterity measured with Purdue pegboard at CE 1, 2, and 3 are given in Table 2 for the EMLA group and placebo group. In addition, box plots are added for visualization of 2PD data of digit II and QuickDASH data in the EMLA and placebo groups ( Fig. 2 ).

**Table 2:** Clinical evaluation (CE) results

Cohort	Evaluated		Clinical evaluation (CE) timepoint			Statistics	
	Side	Digit	CE 1	CE 2	CE 3	CE 1 vs. CE 2	CE 1 vs. CE 3
**BCTQ** median symptom severity score (SSS) (range: 0–5)							
EMLA			2.3 (1.5–3.9) a	–	2.3 (1–2.8) a	–	n.s.
Placebo			2.7 (1.5–4.5)	–	2.4 (1.2–4.5)	–	*p* < 0.05
**QuickDASH** median activity limitation (range: 0–100)							
EMLA			22.7 (4.5–54.5) a	–	11.4 (2.3–45.5) a	–	*p* = 0.05
Placebo			34.1 (0–86.4)	–	27.3 (0–86.4)	–	n.s.
**Tactile discrimination** median 2PD mm (range)							
EMLA	IL	II	4 (2.5–5)	2.8 (2.2–4.3)	3.7 (2.2–4.3) a	*p* < 0.05	*p* < 0.05
	CL	II	2.8 (2.5–4)	3.1 (2.2–4.3)	2.5 (2.5–4) a	n.s.	n.s.
	IL	V	4.0 (3–4.6)	3.1 (2.8–4.3)	4.0 (2.8–5) a	*p* < 0.05	n.s.
	CL	V	4.0 (2.2–5)	3.4 (2.8–5)	3.4 (2.8–6) a	n.s.	n.s.
Placebo	IL	II	3.4 (2.2–7)	3.4 (2.2–7)	3.1 (2.5–4)	n.s.	n.s.
	CL	II	3.4 (2.2–4)	3.1 (2.2–4)	2.8 (2.2–4)	n.s.	n.s.
	IL	V	4.0 (2.8–8)	4.0 (2.8–9)	3.7 (2.5–5)	n.s.	n.s.
	CL	V	4.0 (2.8–5)	3.7 (2.8–6)	3.7 (2.5–4.6)	n.s.	n.s.
**Purdue pegboard** , median score							
EMLA	IL		14 (9–16) a	–	16 (10–15) b	–	*p* < 0.05
	CL		13 (10–16) a	–	12 (10–15) b	–	n.s.
Placebo	IL		13 (11–18)	–	14 (12–17)	–	n.s.
	CL		14 (12–16)	–	13 (10–15)	–	n.s.

Abbreviations: 2PD, two-point discrimination; BCTDQ, Boston carpal tunnel syndrome questionnaire, CL, contralateral to hand affected by carpal tunnel syndrome; IL, ipsilateral to hand affected by carpal tunnel syndrome; n.s., not statistically significant; QuickDASH, disability of arm, shoulder, and hand questionnaire.

Note: Differences within groups over time are presented as *p* -values (Wilcoxon signed-rank test); no statistically significant differences were found between the EMLA and placebo groups across the different clinical evaluation timepoints (CE 1 to CE 3).

a Missing *n* = 2

b Missing *n* = 3; EMLA, AstraZeneca AB, Södertälje, Sweden.

There were no significant differences in any clinical analyses between the EMLA and placebo groups at baseline or the two clinical follow-up examinations. However, in the EMLA group, tactile discrimination (2PD) improved significantly on the affected side in both digit II and digit V following the first treatment and a significant improvement compared with baseline remained in digit II following 8 weeks of treatment. Dexterity assessed with the Purdue pegboard improved significantly on the affected side between CE 1 and CE 3 as did patient-reported outcome, assessed by QuickDASH following 8 weeks treatment with EMLA. The placebo group did not show any statistically significant improvement over time except for BCTQ between CE 1 and CE 3. ENG parameters did not improve in the cohorts after 8 weeks of treatment.

### Functional MRI

Study participants comprised 24 subjects in MR 1 and 23 subjects in MR 2. Four subjects declined MR 3 and thus fMRI data from all three MR examinations were acquired and evaluated from 20 subjects.

**Fig. 3:**
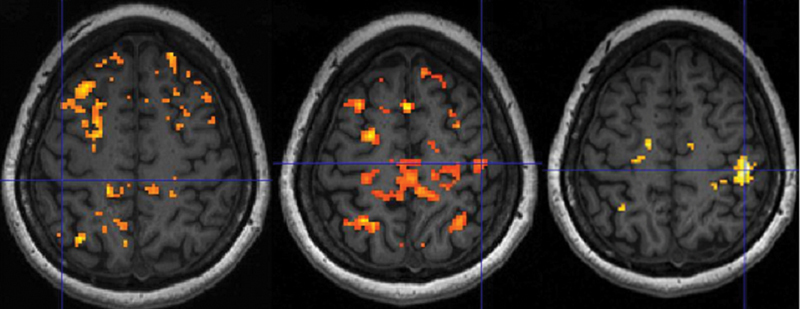
Activation clusters at baseline ( *n* = 24) related to stimulation of median nerve-innervated fingers (digits I and II) of the hand affected by unilateral CTS (left) and of the healthy hand (center). Additionally, activation clusters related to the contrast “digits I and II of the healthy hand” greater than “digits I and II of the affected hand” (right); extent threshold *k* = 0 voxels, *p* < 0.01 (unc.). Crosshairs indicate the local maximum within the primary somatosensory cortex, or corresponding anatomical location if no activation is detected. Neurological presentation displaying right hemisphere to the right.

Activation clusters in the complete cohort (EMLA and placebo, *n* = 24) at baseline (MR 1) for the median nerve-innervated digits I and II are given in Table 3 and illustrated in Fig. 3 for the healthy and CTS-affected hand. Stimulation of digit V, innervated by the ulnar nerve, did not render fMRI activation cluster differences between the two hands (data not shown). Contrasts evaluating potential activation differences between the EMLA and placebo group (EMLA > placebo or placebo > EMLA) at baseline regarding stimulation of the healthy hand or CTS-affected hand, respectively, did not render any statistical differences or activation clusters. The exception was a minimal cluster with coordinates 30, −30, 52 and a cluster size of 4 voxels, *p* = 0.01 (unc.) for the contrast “placebo > EMLA” and stimulation of the healthy hand versus rest (data not shown).

**Table 3:** fMRI results in patients with unilateral carpal tunnel syndrome (CTS) using a task-based sensory stimulation paradigm

Cohort	MR exam	Stimulated hand	Contrast	Cluster maximum coordinates			Cluster sizes a	Illustration presented in
				***x***	***y***	***z***		
**Baseline**								
All patients	MR 1	CTS affected	Activation CTS-affected hand	–	–	–	–	Fig. 2 , left
All patients	MR 1	Healthy	Activation healthy hand	42	−20	58	45 b	Fig. 2 , center
All patients	MR 1	Healthy and CTS affected	Activation healthy hand > activation CTS-affected hand	44	−28	54	293	Fig. 2 , right
All patients	MR 1	Healthy and CTS affected	Any contrast between placebo and EMLA groups	–	–	–	–	–
**90-minute treatment effect**								
EMLA	MR 1 and 2	CTS affected	Activation MR 2 > MR 1	−29	−31	60	111	Fig. 3 , left
EMLA	MR 1 and 2	Healthy and CTS affected	Activation MR 2 > MR 1 and activation CTS affected hand > activation healthy hand	−32	−30	58	31	Fig. 3 , center
EMLA	MR 1 and 2	Healthy and CTS affected	Activation MR 2 > MR 1 and activation healthy hand > activation CTS affected hand	–	–	–	–	Fig. 3 , right
Placebo	MR 1 and 2	Healthy and CTS affected	Any contrast between MR 2 and MR 1	–	–	–	–	–
All patients	MR 1 and 2	Healthy and CTS affected	Any contrast between placebo and EMLA groups	–	–	–	–	–
**8-week treatment effects**								
All patients	MR 3	Healthy and CTS affected	Activation EMLA > placebo and activation CTS affected hand > activation healthy hand	−46	−30	56	17	Fig. 4
All patients	MR 1 and 3	Healthy and CTS affected	Any contrast between MR 1 and MR 3	–	–	–	–	–

Note: Results represent stimulation of median nerve-innervated digits I and II of the healthy and CTS affected hand, respectively, at baseline (MR 1) and after 90 minutes (MR 2) and 8 weeks (MR 3) of treatment with either EMLA or placebo supplemented by additional sensory training. Cluster details are presented for activation clusters located in the primary somatosensory cortex associated with the hand.

a Only cluster sizes > 5 voxels listed.

b Activation includes three local cluster maxima and coordinates and size given represent the cluster presented in Fig. 2 and located closest to the hand area in the primary somatosensory cortex.

**Fig. 4:**
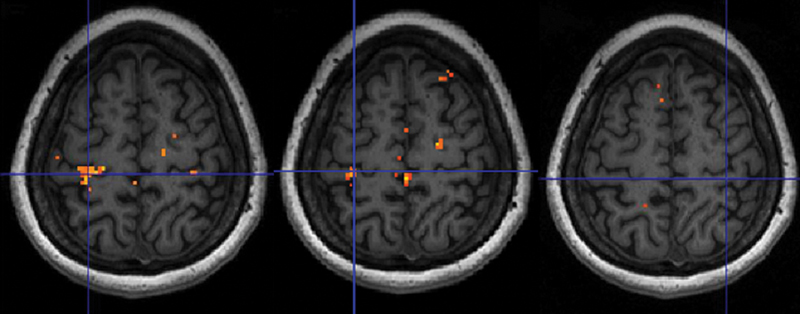
Illustration of cerebral activation after 90-minute EMLA treatment. Stimulation of digits I and II of the affected hand in patients treated with EMLA and contrasting “activation posttreatment (MR 2)” greater than “activation at baseline (MR 1)” shows a distinct activation cluster representing treatment response (left panel). Contrasting “activation MR 2” greater than “activation MR 1” shows activation clusters representing the contrast “activation of hand affected by CTS” greater than “activation of healthy hand” (center panel) but no activation clusters for the contrast “activation healthy hand” greater than “activation of hand affected by CTS” (right panel). The placebo group did not exhibit any treatment effects in comparable analyses for the hand affected by CTS (data not shown). Paired *t* -test, extent threshold *k* = 0 voxels, *p* < 0.01 (unc.). Crosshairs indicate the local maximum, or anatomical location of the primary somatosensory hand area. Neurological presentation displaying right hemisphere to the right.

After 90 minutes of treatment (MR 2) with EMLA or placebo, mean activation in the hand area of the primary somatosensory cortex, following stimulation of median nerve-innervated digits I and II of the hand affected by CTS, increased compared with baseline (MR 1) in the group treated with EMLA ( Fig. 4 , Table 3 ). No change was seen related to stimulation of digits I and II of the healthy hand or in the placebo group or between groups (data not shown).

**Fig. 5:**
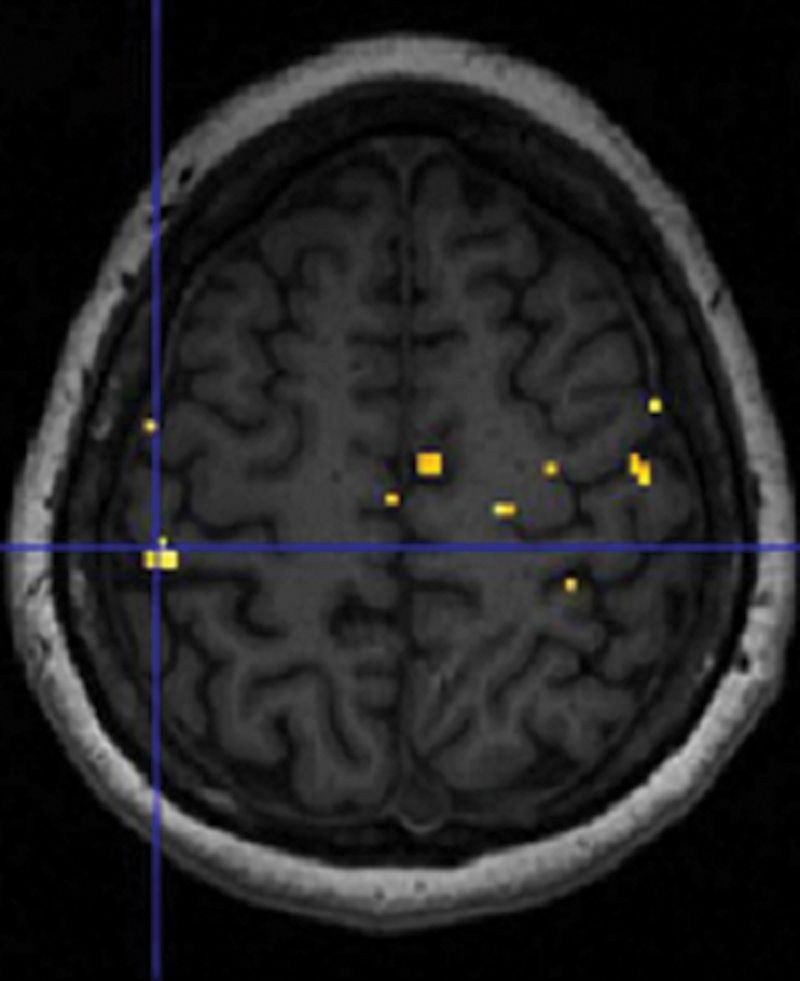
After 8 weeks of treatment, an activation cluster was observed when contrasting “activation in patients treated with EMLA” greater than “activation in patients treated with placebo” and “activation to stimulation of the hand affected by CTS” greater than “activation of the healthy hand”; *t* -test, extent threshold *k* = 0 voxels, *p* < 0.01 (unc.). Crosshair indicates the local maximum. Neurological presentation displaying right hemisphere to the right.

Following 8 weeks of treatment (MR 3), stimulation of digits I and II of the “hand affected by CTS” in contrast to the “healthy hand” showed increased cortical activation in the hand area of the primary somatosensory cortex when contrasting “patients treated with EMLA” against “patients treated with placebo” ( Fig. 5 , Table 3 ). No statistically significant differences could be seen when contrasting MR 3 versus MR 1.

## Discussion

Cutaneous stimulation of median nerve-innervated fingers in patients with unilateral CTS resulted in decreased activation in the contralateral primary somatosensory cortex (S1). Furthermore, treatment with cutaneous forearm anesthesia, on the side with CTS, resulted in larger activation in the contralateral S1 at the 90-minute follow-up, and compared with the placebo group at the 8-week follow-up, a slight improvement in tactile discrimination.

From studies on primates and humans, it is well known that a median nerve that has been transected and subsequently repaired results in substantial functional and structural changes in the brain. 12 13 40 The mechanism behind these changes is thought to be the altered afferent signal pattern seen in the injured nerve. In humans, there is evidence that the clinical recovery of sensory function following median nerve repair is linked to cerebral adaptations in both brain hemispheres. 12 CTS, where the median nerve is compressed at the wrist, also results in a changed afferent signal to the brain and a limited number of studies have suggested that CTS results in cerebral changes 14 16 41 ; however, none of these studies have studied unilateral CTS.

Studies where fMRI has been used to assess cerebral activation following median nerve injury have shown a larger activation in the contralateral S1 representing a disruption of the normal somatotopy. 12 40 42 43 Previous studies using different neuroimaging techniques such as magnetoencephalograpy, 44 45 46 resting-state fMRI, 16 and fMRI 15 in patients with bilateral CTS have shown conflicting results and often conclude that CTS results in functional and structural changes in the brain. 47 48 One study, using fMRI in patients with bilateral CTS, found more extensive and stronger contralateral sensorimotor activation compared with controls. 14 We found that stimulation of median nerve-innervated fingers in the hand with CTS resulted in a smaller activation in the contralateral S1, compared with stimulation of the healthy hand. The smaller activation seen in this study is likely explained by a slower afferent nerve signal with a lower amplitude and that part of the axons in the median nerve probably does not transmit afferent signals at all. The differences in results between the current study and the one by Napadow et al 14 might be explained by the use of different fMRI paradigms. We choose not to normalize the intensity of sensory stimulation; all participants in this study had a normal discriminatory sensation and were compared at the same intensity of tactile stimulation (1 Hz, 2.5 bar). On the other hand, Napadow et al 14 applied electrical stimulation in correlation with the subjects' pain threshold. It is possible that this adaptation of the stimulation could yield a larger activation than seen in our study. Furthermore, when the sensory stimulation is substantially increased, it may result in crosstalk between sensory and pain pathways, where a stronger stimulus conceivably, in addition to activating sensory pathways also, could activate pain pathways more and thus increase activation. 49 50

The current findings, showing a smaller activation in the contralateral S1 in patients with CTS, are in line with previous studies. 16 Further studies including patients with more severe CTS are needed to assess if the extent of cerebral changes depends on the severity of entrapment. Following a CTR, many patients experience improvement of symptoms, but it is well known that ENG can remain pathologic long after surgery, whereas symptoms often improve immediately after surgery. This implies that part of the improvement may be related to cerebral adaptations. It is of further clinical interest why some patients with CTS do not experience improvement after CTR. Residual symptoms following a CTR may partly be explained by maladaptive plasticity and rehabilitation should be considered as an initial treatment alternative in such cases before additional surgery is considered.

The concept of guided plasticity, in the form of cutaneous forearm anesthesia, has been tested on patients with median and ulnar nerve injuries 12 and in patients with vibration-induced neuropathy, showing improved clinical results and larger activation in the S1 following anesthesia. This suggests that improved sensory function in the hand following cutaneous forearm anesthesia is based on cerebral plasticity. The exact mechanism behind this plasticity was outside the scope of the current study. However, research on cerebral response following deafferentation suggests that reorganization following permanent as well as transient deafferentation is mediated by two processes: (1) a decreased inhibition mediated by decreased GABA (gamma-aminobutyric acid) levels in the deafferented areas and (2) sprouting of axons from cortical areas adjacent to the deafferented area. In addition, GABAergic activity can increase in the brain following acute deafferentation. 51 It is unlikely that changes in the peripheral nerve lead to improved sensibility without activating the neural correlate for sensibility, i.e., the hand area in the primary somatosensory cortex (S1). Thus, the most plausible explanation for the increased activation in the S1 and the improved sensibility in the fingers is cutaneous deafferentation.

One prior study assessed acupuncture for treatment for CTS, 20 showing that 8 weeks of acupuncture improved peripheral and cerebral neurophysiological outcomes. It is not clear whether plasticity is guided in acupuncture since mechanisms enabling acupuncture are not fully understood. However, two possible mechanisms are put forward, the gate theory 52 and another theory concerning the release of CNS analgesic substances. 53 A systematic review on acupuncture showed that acupuncture could increase blood flow in certain brain areas such as the S1 and cognitive areas. 54 However, a previous Cochrane analysis concluded that acupuncture has little or no effects in the short term on symptoms in CTS patients. 53

The majority of participants in this study had a normal sensory function and thus the potential for clinical changes after 8 weeks of treatment is small (i.e., ceiling effect). Both groups showed a tendency to improvement in 2PD in tested fingers (digits II and V) over time. This is most likely the result of the sensory training program given to all participants. Interestingly, only the median nerve-innervated finger in the EMLA group showed significant improvement in 2PD over 8 weeks. Together with fMRI showing a larger activation in the contralateral S1 following EMLA treatment, this suggests a cerebral effect of the guided plasticity treatment. However, the improvements in both 2PD and BCTQ are small and likely not clinically relevant. We can only speculate that patients with more severe CTS, and thus more impaired tactile discrimination, might benefit more from EMLA treatment.

The present study corroborates in parts the results from a study 20 where acupuncture was used, showing larger activation in the contralateral S1 and improved tactile discrimination in median nerve-innervated fingers as well as improvement in how patients rated their subjective symptoms following 8 weeks of treatment using guided plasticity. Furthermore, Maeda et al 20 showed that acupuncture improved nerve conduction, whereas the present study could not detect any changes in nerve conduction over time.

Guided plasticity, in the form of cutaneous anesthesia of the forearm, may have a role in the treatment of patients with CTS. However, in the majority of patients, symptoms of CTS are caused by local entrapment of the median nerve and these patients benefit from a CTR. On the other hand, patients operated with adequate decompression via CTR but without complete symptom relief might benefit from treatment with guided plasticity. In such patients, symptoms may be caused by injury to axons in the median nerve and/or cerebral maladaptation. Future prospective studies are needed to assess the role of cutaneous forearm anesthesia on patients with residual symptoms following CTR and median neuropathy due to other reasons than CTS.

This study had some limitations. Patients were not recruited consecutively. CTS is a common disease with high potential of symptom release after surgery leaving patients reluctant to undergo a preoperative treatment study for 8 weeks. In addition, very few patients met the inclusion criteria of unilateral CTS, both clinically and neurographically. We chose unilateral CTS to allow for each patient to be his/her internal control. An additional limitation is differences in age and symptoms at inclusion. The median age in the EMLA group was 55 years and in the placebo group 43 years. However, individuals aged 40 to 55 years show similar sensibility and electroneurographic parameters. 55 Differences in sensibility and electroneurographic parameters are first evident when comparing individuals aged 40 to 55 with those aged 60 and above. QuickDASH scores were lower in the EMLA group compared with the placebo group. However, more patients had moderate CTS according to Padua in the EMLA group compared with the placebo group. Additionally, the more sensitive instrument BCTQ, which is specific for CTS, did not show any clinical difference between the groups at inclusion. Thus, it is not likely that the small differences between groups in age and symptoms at inclusion are of clinical significance and affect the results. The stimulation equipment used in this study has been used in several studies before and shown good reliability. 34 However, as the stimulation equipment was attached to the fingers during the complete experiment, this might result in some constant touch experience decreasing the bold effect considering the block design of the fMRI experiment.

## Conclusions

Stimulation of median nerve-innervated fingers in patients with unilateral CTS results in cerebral changes with a smaller-than-normal activation in the contralateral S1. Cutaneous forearm anesthesia on the side with CTS results in larger activation in S1, suggesting recruitment of more neurons in line with the theory of guided plasticity treatment and a slight improvement in sensory function. Further studies are needed to better understand how cerebral changes affect the symptoms in patients with CTS, and also to describe the role of treatment strategies where brain plasticity is guided to improve function in patients with CTS and other neuropathies.
